# Genome-wide association study in Chinese Holstein cows reveal two candidate genes for somatic cell score as an indicator for mastitis susceptibility

**DOI:** 10.1186/s12863-015-0263-3

**Published:** 2015-09-15

**Authors:** Xiao Wang, Peipei Ma, Jianfeng Liu, Qin Zhang, Yuan Zhang, Xiangdong Ding, Li Jiang, Yachun Wang, Yi Zhang, Dongxiao Sun, Shengli Zhang, Guosheng Su, Ying Yu

**Affiliations:** Key Laboratory of Animal Genetics, Breeding and Reproduction, Ministry of Agriculture of China, National Engineering Laboratory for Animal Breeding, College of Animal Science and Technology, China Agricultural University, 100193 Beijing, People’s Republic of China; Department of Molecular Biology and Genetics, Center for Quantitative Genetics and Genomics, Aarhus University, DK-8830 Tjele, Denmark

**Keywords:** Genome-wide association study, EBVs of somatic cell scores, Chinese Holstein cows, Mixed model based single locus regression analysis, Mastitis susceptibility

## Abstract

**Backgrounds:**

Bovine mastitis is a typical inflammatory disease causing seriously economic loss. Genome-wide association study (GWAS) can be a powerful method to promote marker assistant selection of this kind of complex disease. The present study aimed to analyze and identify single nucleotide polymorphisms (SNPs) and candidate genes that associated with mastitis susceptibility traits in Chinese Holstein.

**Results:**

Forty eight SNPs were identified significantly associated with mastitis resistance traits in Chinese Holstein cows, which are mainly located on the BTA 14. A total of 41 significant SNPs were linked to 31 annotated bovine genes. Gene Ontology and pathway enrichment revealed 5 genes involved in 32 pathways, in which, *TRAPPC9* and *ARHGAP39* genes participate cell differentiation and developmental pathway together. The six common genome-wide significant SNPs are found located within *TRAPPC9* and flanking *ARHGAP39* genes.

**Conclusions:**

Our data identified the six SNPs significantly associated with SCS EBVs, which suggest that their linked two genes (*TRAPPC9* and *ARHGAP39*) are novel candidate genes of mastitis susceptibility in Holsteins.

**Electronic supplementary material:**

The online version of this article (doi:10.1186/s12863-015-0263-3) contains supplementary material, which is available to authorized users.

## Background

Bovine mastitis is one of the most typical inflammatory diseases causing seriously economic loss in modern dairy farms and quality problems of dairy food worldwide [1]. Since the heritability of mastitis is low, genetic improvement on anti-mastitis by traditional selection is not very effective [2]. Moreover, it is not easy to measure mastitis in field scale. Somatic cell count (SCC) or log transformed SCC (somatic cell score, SCS) have relatively higher heritability compared to mastitis and are used as the first trait to improve mastitis resistance [3]. In addition, to avoid uncertain influences such as farms, seasons, sires and etc., estimated breeding values (EBVs) of somatic cell scores (SCSs) were normally used as pseudo-phenotypes of mastitis related traits in dairy cattle. Genome-wide association study (GWAS) is widely considered a potential method to promote marker assisted selection of mastitis related traits based on single nucleotide polymorphism (SNP) [4].

The previous GWAS for mastitis susceptibility showed multifarious results in different Holstein populations. Family-based association tests such as single locus regression analysis and transmission disequilibrium test have the robust advantage to population heterogeneity [5]. In 2011, Sodeland’s group detected QTLs for clinical mastitis on *Bos taurus* autosome (BTA) 2, 6, 14, and 20 in Norwegian red cattle [6]. In 2012, Meredith *et al*. reported that 9 SNPs located on BTA 6, 10, 15 and 20 were significantly associated with SCSs in Holstein sires and cows [7]. The same year, Wijga *et al*. [8] reported that SNPs relevant to log transformed lactation-average somatic cell scores or the standard deviation of test-day somatic cell score were mainly located on BTA 4, 6 and 18. In addition, strong associations of SNPs with clinical mastitis and SCS were reported on bovine BTA 6, 13, 14 and 20 in Nordic Holstein cattle by Sahana *et al*. [9]. Recently, GWAS performed in German Holstein cows identified significant SNPs on BTA 6, 13, 19 and X [10]. The studies in US Holstein dairy cows have shown that genetic variants on BTA 2, 14, 20 have impacts on clinical mastitis. The identified region on BTA 14 contains lymphocyte-antigen-6 complex (*LY6*) including *LY6K*, *LY6D*, *LYNX1*, *LYPD2*, *SLURP1*, *PSCA* genes in regulating the major histocompatibility complex [11]. The studies in Chinese population containing Chinese Holstein, Sanhe cattle and Chinese Simmental have analyzed that *TLR4* gene (Toll-like receptor 4) and *BRCA1* gene (Breast cancer 1) have the significant association with SCS [12, 13]. Even though many studies have identified significant SNPs, only one SNP (BTA-77077-no-rs, Position: 85527109) on BTA 6 was identical in the reports of Sahana *et al*. [9] and Abdel-Shafy *et al* [10]. These results implied that the significant SNPs associated with mastitis traits were not identified consistently and should be confirmed and validated in different Holstein populations.

In order to detect functional candidate genes for mastitis-related traits, GWAS was conducted with mixed model based single locus regression analysis (MMRA) in Chinese Holstein populations. Six common SNPs were identified by MMRA and two linked genes were disclosed with significant effects on mastitis-related traits in Chinese Holstein populations.

## Results

### Significant SNPs associated with SCSs EBVs

**Fig. 1:**
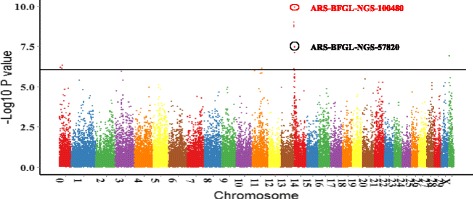
Manhattan plots of genome-wide association for SCS EBVs

The –log_10_*P* of all tested SNPs for SCS EBVs with MMRA is shown in Fig. 1. The significant SNPs associated with SCS EBVs were mainly located on BTA 14.

The genomic association SNPs detected by MMRA were presented in Table 1. In total, 48 significant SNPs on chromosome level were detected including 13 SNPs on genome level. As shown in Table 1, 41 out of 48 SNPs were located within or near 31 known genes.

**Table 1:** Chromosome-wide significant SNPs for SCS EBVs

SNP name	Chr.	Position(bp)	Nearest genes^a^	Distance(bp)	*P-*values
ARS-BFGL-NGS-32524	0^b^	NA	NA	NA	4.79E-07
ARS-BFGL-NGS-18858	0^b^	NA	NA	NA	7.00E-07
BFGL-NGS-114657	0^b^	NA	NA	NA	6.61E-07
ARS-BFGL-NGS-91137	0^b^	NA	NA	NA	1.48E-05
ARS-BFGL-NGS-60730	0^b^	NA	NA	NA	3.41E-05
ARS-BFGL-NGS-103637	1	59166287	*SIDT1*	within	4.13E-06
ARS-BFGL-NGS-2950	1	84528381	*MAGEF1*	152817	1.71E-05
Hapmap42708-BTA-86534	3	50852627	*RWDD3*	596180	1.15E-06
ARS-BFGL-NGS-55261	3	2281390	*ILDR2*	212007	1.91E-05
Hapmap32072-BTA-142491	4	106961853	*TBXAS1*	16314	1.13E-05
Hapmap51299-BTA-73473	5	47059558	*RAB3A*	86426	7.44E-06
ARS-BFGL-NGS-104108	5	71073538	*IGF1*	52675	1.48E-05
BTB-01491979	8	107025584	*LOC534155*	130161	2.22E-05
BTB-00391421	9	50410127	*GRIK2*	within	1.08E-05
Hapmap51481-BTA-67522	9	49607152	*GRIK2*	375591	1.51E-05
BTB-00391456	9	50434277	*GRIK2*	within	2.49E-05
ARS-BFGL-NGS-3540	11	68044963	*C1D*	359533	6.99E-07
Hapmap39693-BTA-85506	11	15115923	*MEMO1*	51033	1.09E-06
ARS-BFGL-BAC-14940	11	67828555	*ETAA1*	173499	1.42E-06
Hapmap31821-BTA-156670	13	4956832	NA	NA	1.14E-05
ARS-BFGL-NGS-100480	14	2607583	*TRAPPC9*	within	1.24E-10
ARS-BFGL-NGS-4939	14	443937	*ARHGAP39*	258178	9.97E-10
ARS-BFGL-NGS-107379	14	679600	*ARHGAP39*	460	1.63E-09
ARS-BFGL-NGS-57820	14	236532	*ARHGAP39*	50773	1.97E-09
ARS-BFGL-NGS-56327	14	2580414	*TRAPPC9*	within	3.29E-08
UA-IFASA-5306	14	2711615	*TRAPPC9*	within	3.64E-08
UA-IFASA-9288	14	2201870	*PTK2*	within	8.29E-08
ARS-BFGL-NGS-18365	14	741867	*MAPK15*	111034	2.77E-06
BFGL-NGS-113575	14	2484499	*TRAPPC9*	within	1.08E-05
BFGL-NGS-111902	14	65409003	*TSPYL5*	370903	1.86E-05
ARS-BFGL-NGS-104701	16	56834152	*GLRX*	191565	2.73E-05
ARS-BFGL-BAC-33744	19	34229778	*NCOR1*	within	4.14E-05
ARS-BFGL-NGS-44441	20	13114376	*CD180*	31009	3.59E-06
ARS-BFGL-NGS-106084	21	57855394	*ITPK1*	180441	5.48E-06
ARS-BFGL-NGS-61681	21	30197672	*CHRNA7*	499598	5.65E-06
ARS-BFGL-NGS-41216	21	25613731	*BCL2A1*	141178	1.12E-05
ARS-BFGL-NGS-7344	21	42702373	*G2E3*	521371	1.54E-05
ARS-BFGL-NGS-39846	27	36421058	*PLEKHA2*	12209	5.81E-06
ARS-BFGL-NGS-71055	27	37589834	*IDO1*	198717	8.77E-06
ARS-BFGL-NGS-29650	27	36946859	*IDO1*	431343	1.55E-05
ARS-BFGL-NGS-108861	27	37445592	*IDO1*	54475	4.96E-05
UA-IFASA-6255	28	41464821	*BMPR1A*	within	3.80E-05
BTB-01016631	29	28085086	*SAA2*	355019	5.76E-06
ARS-BFGL-NGS-12475	29	21777960	*LUZP2*	47926	9.44E-06
BTB-01337464	29	29072341	NA	NA	3.04E-05
Hapmap56639-rs29021780	X	2460976	*GRIA3*	within	1.34E-07
Hapmap57012-rs29019338	X	12135331	*F9*	821885	2.85E-06
ARS-BFGL-NGS-94205	X	2348904	*GRIA3*	within	8.47E-05

NA: not available

^a^Derived from UCSC Genome Bioinformatics (http://genome.ucsc.edu/cgi-bin/hgBlat?command=start)

^b^These SNPs are not assigned to any chromosomes and noted as “0”

In the thirteen genome-wide significant SNPs, ARS-BFGL-NGS-100480 was located within *TRAPPC9* gene (trafficking protein particle complex 9) on BTA 14 and showed lowest *P*-values of 1.24E-10. Two other significant SNPs, ARS-BFGL-NGS-56327 and UA-IFASA-5306 located within *TRAPPC9* gene, were detected with *P*-values of 3.29E-08, and 3.64E-08, respectively. In addition, three other significant SNPs were identified linked with *ARHGAP39* gene (Rho GTPase activating protein 39) (Table 2).

**Table 2:** Genome-wide significant SNPs with genome annotations

SNP name	Chr.	Nearest genes^a^			*P-*values
		Name	Distance(bp)	Full name	
ARS-BFGL-NGS-32524	0^b^	NA	NA	NA	4.79E-07
ARS-BFGL-NGS-18858	0^b^	NA	NA	NA	7.00E-07
BFGL-NGS-114657	0^b^	NA	NA	NA	6.61E-07
ARS-BFGL-NGS-3540	11	*C1D*	359533	C1D nuclear receptor corepressor	6.99E-07
Hapmap39693-BTA-85506	11	*MEMO1*	51033	mediator of cell motility 1	1.09E-06
ARS-BFGL-NGS-100480	14	*TRAPPC9*	within	trafficking protein particle complex 9	1.24E-10
ARS-BFGL-NGS-4939	14	*ARHGAP39*	258178	Rho GTPase activating protein 39	9.97E-10
ARS-BFGL-NGS-107379	14	*ARHGAP39*	460	Rho GTPase activating protein 39	1.63E-09
ARS-BFGL-NGS-57820	14	*ARHGAP39*	50773	Rho GTPase activating protein 39	1.97E-09
ARS-BFGL-NGS-56327	14	*TRAPPC9*	within	trafficking protein particle complex 9	3.29E-08
UA-IFASA-5306	14	*TRAPPC9*	within	trafficking protein particle complex 9	3.64E-08
UA-IFASA-9288	14	*PTK2*	within	PTK2 protein tyrosine kinase 2	8.29E-08
Hapmap56639-rs29021780	X	*GRIA3*	within	glutamate receptor, ionotrophic, AMPA 3	1.34E-07

*NA* not available

^a^Derived from UCSC Genome Bioinformatics (http://genome.ucsc.edu/cgi-bin/hgBlat?command=start)

^b^These SNPs are not assigned to any chromosomes and noted as “0”

### Linkage disequilibrium (LD) blocks of the significant SNPs on BTA 14

**Fig. 2:**
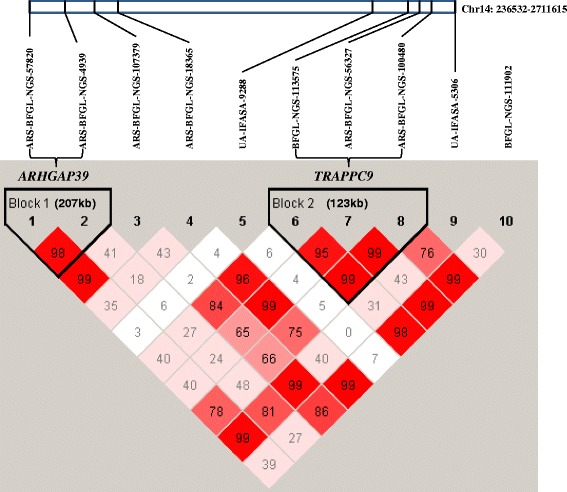
Linkage disequilibrium (LD) pattern for 10 significant SNPs on BTA 14. Solid line triangles refer to linkage disequilibrium (LD). One square refers to LD level (r2) between two SNPs and the squares are colored by D’/LOD standard scheme (LOD is the logarithm of likelihood odds ratio and the reliable index to measure D’). D’/LOD standard scheme is that red refers to LOD > 2, D’ = 1; pink refers to LOD > 2, D’ < 1; blue refers to LOD < 2, D’ = 1; white refers to LOD < 2, D’ < 1

Linkage disequilibrium analysis for the total ten significant SNPs on BTA 14 showed two LD blocks (Fig. 2). Two significant SNPs (ARS-BFGL-NGS-57820 and ARS-BFGL-NGS-4939) in the block 1 were located on the upstream of *ARHGAP39* gene, and three significant SNPs (BFGL-NGS-113575, ARS-BFGL-NGS-56327 and ARS-BFGL-NGS-100480) in the block 2 were located within *TRAPPC9* gene.

### Two candidate genes for mastitis-related traits

**Fig. 3:**
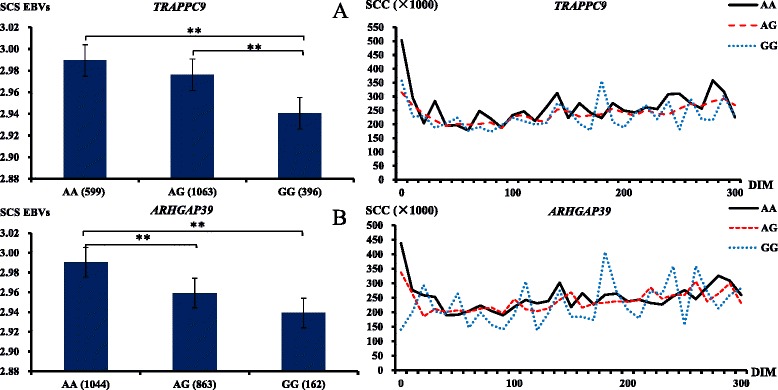
The SCS EBVs and curves of SCC in different genotypes of *TRAPPC9* and *ARHGAP39* genes. **refers to *P* < 0.001

*TRAPPC9* and *ARHGAP39* genes (each contains three significant SNPs on genome level) identified by MMRA can be considered potential candidate genes for mastitis-related traits. To decipher the effect of each genotype in each potential candidate gene on mastitis-related traits, the SCS EBVs of the cows with three genotypes were compared. As shown in the left panel of the Fig. 3, the cows with genotype AA in the two genes all owned significant higher SCS EBVs compared to the other genotypes (*P* < 0.001). These results appropriately confirmed the two genes (*TRAPPC9* and *ARHGAP39*) as potential candidate genes for SCS EBVs. The right panel of the Fig. 3 showed the average original phenotypic SCC of the cows with three genotypes for each gene fluctuated with the days in milk (DIM). It was displayed that the cows with genotype AA had a tendency of higher SCC along DIM than the other two genotypes for the two genes especially for *TRAPPC9* gene (Fig. 3).

### Gene ontology and pathway enrichment for the significant SNPs on genome level

**Fig. 4:**
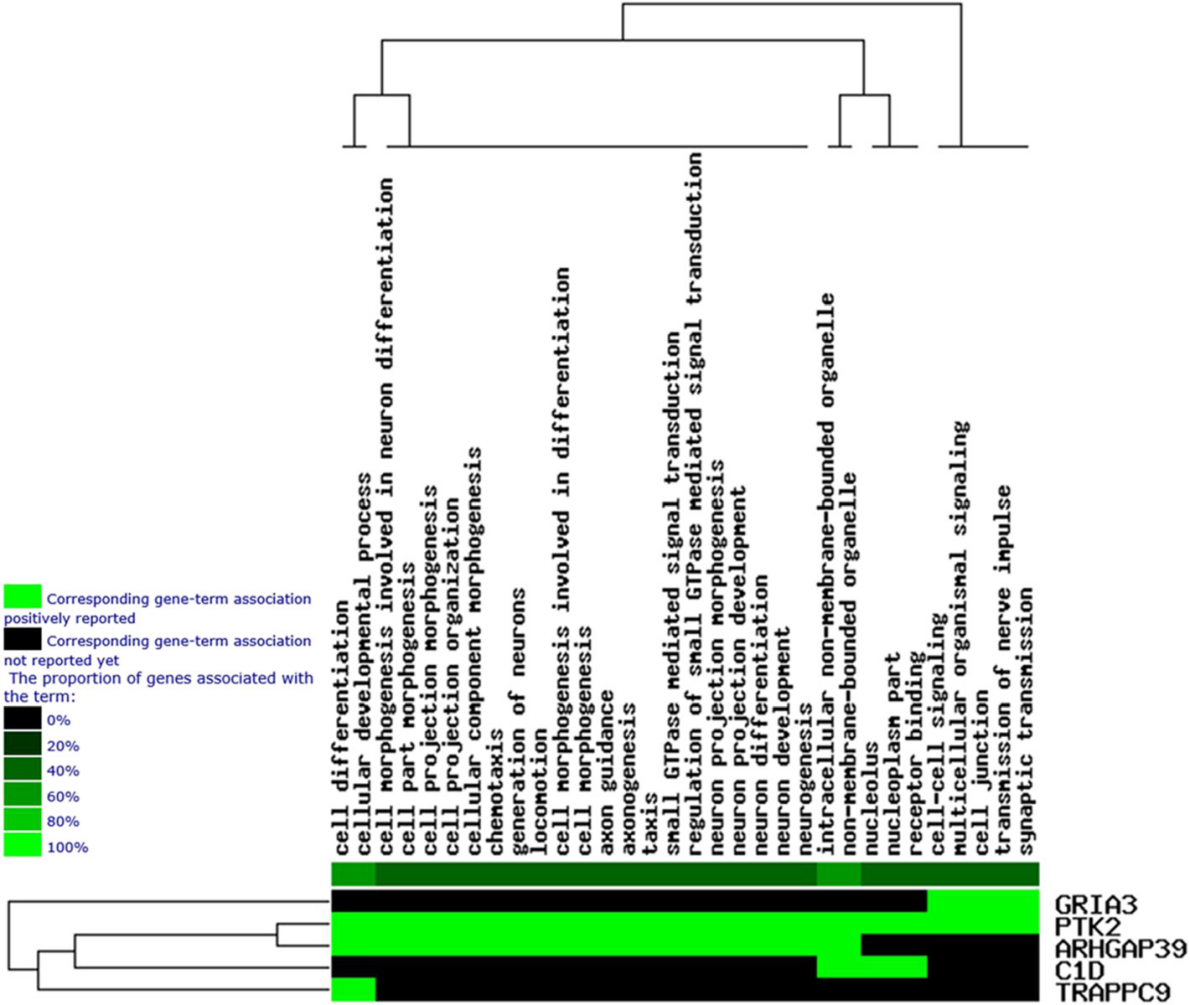
The cluster result of GO analysis

Through the Gene Ontology (GO) analysis of GenCLiP 2.0 (http://ci.smu.edu.cn/GenCLiP2.0/analysis.php?random=new), we found that 5 genes perform mainly functions in 32 pathway terms presented in Table 3 and Fig. 4. Through enrichment of five genes, *ARHGAP39* gene can totally participate 24 pathway terms including two pathway terms combined with *TRAPPC*9 gene (GO:0030154 and GO:0048869), which influence cell differentiation or cellular developmental process.

**Table 3:** Results of GO analysis^a^

Pathway	Hit	Total	*P*-Value	*Q*-Value	Gene	List
axon guidance	2	360	0.004	0.357	*ARHGAP39;PTK2*	GO:0007411
Taxis	2	608	0.012	0.165	*ARHGAP39;PTK2*	GO:0042330
regulation of small GTPase mediated signal transduction	2	425	0.006	0.247	*ARHGAP39;PTK2*	GO:0051056
Axonogenesis	2	517	0.009	0.241	*ARHGAP39;PTK2*	GO:0007409
cell morphogenesis involved in neuron differentiation	2	568	0.010	0.217	*ARHGAP39;PTK2*	GO:0048667
neuron projection morphogenesis	2	576	0.011	0.179	*ARHGAP39;PTK2*	GO:0048812
neuron projection development	2	703	0.016	0.101	*ARHGAP39;PTK2*	GO:0031175
Chemotaxis	2	608	0.012	0.142	*ARHGAP39;PTK2*	GO:0006935
small GTPase mediated signal transduction	2	676	0.015	0.135	*ARHGAP39;PTK2*	GO:0007264
cell projection morphogenesis	2	689	0.015	0.126	*ARHGAP39;PTK2*	GO:0048858
cell part morphogenesis	2	701	0.016	0.118	*ARHGAP39;PTK2*	GO:0032990
cell morphogenesis involved in differentiation	2	709	0.016	0.095	*ARHGAP39;PTK2*	GO:0000904
neuron development	2	813	0.021	0.096	*ARHGAP39;PTK2*	GO:0048666
cell projection organization	2	949	0.028	0.116	*ARHGAP39;PTK2*	GO:0030030
cell morphogenesis	2	968	0.029	0.115	*ARHGAP39;PTK2*	GO:0000902
neuron differentiation	2	1008	0.031	0.118	*ARHGAP39;PTK2*	GO:0030182
cellular component morphogenesis	2	1026	0.032	0.117	*ARHGAP39;PTK2*	GO:0032989
generation of neurons	2	1088	0.036	0.120	*ARHGAP39;PTK2*	GO:0048699
Neurogenesis	2	1156	0.040	0.120	*ARHGAP39;PTK2*	GO:0022008
Locomotion	2	1282	0.049	0.127	*ARHGAP39;PTK2*	GO:0040011
synaptic transmission	2	702	0.016	0.109	*GRIA3;PTK2*	GO:0007268
multicellular organismal signaling	2	812	0.021	0.101	*GRIA3;PTK2*	GO:0035637
cell junction	2	771	0.019	0.104	*GRIA3;PTK2*	GO:0030054
transmission of nerve impulse	2	791	0.020	0.103	*GRIA3;PTK2*	GO:0019226
cell-cell signaling	2	1135	0.039	0.120	*GRIA3;PTK2*	GO:0007267
Nucleolus	2	628	0.013	0.132	*C1D;PTK2*	GO:0005730
nucleoplasm part	2	862	0.023	0.102	*C1D;PTK2*	GO:0044451
receptor binding	2	1206	0.044	0.125	*C1D;PTK2*	GO:0005102
cell differentiation	3	2754	0.033	0.114	*ARHGAP39;PTK2;TRAPPC9*	GO:0030154
cellular developmental process	3	2928	0.039	0.125	*ARHGAP39;PTK2;TRAPPC9*	GO:0048869
intracellular non-membrane-bounded organelle	3	3104	0.046	0.126	*ARHGAP39;C1D;PTK2*	GO:0043232
non-membrane-bounded organelle	3	3104	0.046	0.122	*ARHGAP39;C1D;PTK2*	GO:0043228

^a^Derived from GenCLiP 2.0 (http://ci.smu.edu.cn/GenCLiP2.0/analysis.php?random=new)

## Discussion

The present study identified significant SNPs and novel candidate genes associated with mastitis-related traits in Chinese Holstein population with mixed model based single marker regression analysis (MMRA). Two genes (*TRAPPC9* and *ARHGAP39*) identified by significant SNPs indicate that they are important candidate genes associated with mastitis-related traits. To our knowledge, this is the first study to decompose the genetic background of mastitis-related traits in Chinese dairy cattle using MMRA assay.

With regards to *TRAPPC9* gene, it was reported that its product NIBP (NIK and IKKβ-binding protein) can enhance cytokine-induced NF-κB signaling pathway through interaction with NIK (NF-κB-inducing kinase) and IKKβ (IκB kinase-β) [14, 15]. In recent studies, *TRAPPC9* gene was considered as candidate gene for autosomal recessive non-syndromic mental retardation [16, 17]. In the present study, the SCS EBVs (2.99) of the cows with AA genotype of SNP (ARS-BFGL-NGS-100480) in *TRAPPC9* gene is significantly higher than the other two genotypes (*P* < 0.001). The similar tendency of the three genotypes was independently proved in a completely different Chinese Holstein population (*n* = 314, our unpublished data). As for *ARHGAP39* gene, it was proved to be function to activate Rho GTPase which is known as new targets in cancer therapy [18]. Therefore, it is clear that the present study screened functional closely related genes to bovine mastitis resistance.

From the reported GWAS based on single locus regression analysis, it is not easy to identify the certain SNPs associated with SCS or mastitis-related traits. As shown in Table 1, 7 significant SNPs located on BTA 14 on whole genomic level (*P* < 1.14E-06) by MMRA in Chinese Holsteins were completely different from all the reported significant SNPs [7, 8], whereas significant SNPs on BTA 14 are consistent with other studies [6, 9–11, 19, 20]. In comparison, one significant SNP UA-IFASA-9288 (BTA 14, Position: 2201870) in Chinese Holstein was close to (147413 bp) the SNP ARS-BFGL-NGS-107379 (Position: 2054457) which was identified in Nordic Holstein [9]. However, Tiezz *et al*. [11] identified a region associated with clinical mastitis from 2,574,909 to 3,137,184 bp on BTA 14 which contains three genome-wide significant SNPs (ARS-BFGL-NGS-100480, ARS-BFGL-NGS-56327 and UA-IFASA-5306) covered by *TRAPPC9* gene in this study. These GWAS studies suggest that mastitis-related traits as low heritable polygenetic traits are mainly controlled by multiple loci which distributed across the whole genome and each with relatively small genetic effect.

Although SCS is continuous trait which normally used as important indicator of mastitis, it is usually unstable and easily influenced by environment [21, 22]. Therefore, to disease indicator trait, current strategy has changed to performing association studies in cases and controls test [23], because of mastitis resistance or susceptibility can be considered as threshold traits [2]. In the current another study, we defined that the left and right parts of the population with half/one standard deviation of SCS EBVs were mastitis susceptibility group (case) and healthy group (control), respectively, and analyzed the two groups with ROADTRIPs (Robust Association-Detection Test for Related Individuals with Population Substructure) (version 1.2) (http://faculty.washington.edu/tathornt/software/ROADTRIPS2/) using bovine 54 k SNPs information. Although the decreased population size and increasing bias affect the testing power of the case-control association assay, we also have found two significant SNPs linked to two genes (*TRAPPC9* and *ARHGAP39*) by ROADTRIPs of case-control test compared with MMRA results, which strongly suggest that these genes are novel candidate genes for mastitis traits.

The genes closed to or covered significant SNPs were further subjected to bioinformatics analysis. Results from Gene Ontology (GO) analysis (Table 3) indicated that *TRAPPC9*, *ARHGAP39* and *PTK2* genes play a role in regulation of cell differentiation (GO: 0030154, *P* = 0.033) or developmental process (GO: 0048869, *P* = 0.039). From the cluster result of GO analysis (Fig. 4), we found that *ARHGAP39* and *PTK2* genes are mostly close genes, which participate 24 pathway terms. However, *TRAPPC9* gene has less result in GO analysis, thus the related pathways are needed to do further functional analysis.

## Conclusions

Although lower detecting power exists in SCS EBVs and other mastitis resistance traits, results consistently support that the significant SNPs are mainly located on the BTA 14 in the Chinese Holstein cows. *TRAPPC*9 and *ARHGAP39* genes reveal the two novel candidate genes associated with mastitis resistant traits in dairy cattle.

## Methods

### Ethics statement

All protocols for collection of the blood sample of experimental cows were reviewed and approved by the Institutional Animal Care and Use Committee (IACUC) at China Agricultural University.

### Animals and phenotype

A total of 2,093 cows from 14 sires were collected to construct the study population. The number of daughters of 14 sires range from 83 to 358 with an average of 150. Although the 14 sires were genotyped, they were not used in the association study in order to avoid double use of daughters’ information. These daughters were from 15 Holstein cattle farms in Beijing, China. No specific permissions were required for these locations/activities.

As closely following normal distribution, somatic cell scores (SCSs) are calculated from SCCs as (log_2_ (SCC/100,000) + 3). To avoid environment influence, EBVs of SCSs were provided as the phenotypes in the GWAS. These EBVs were obtained based on a multiple trait random regression test-day model [24] using the software RUNGE provided by Canadian Dairy Network (CDN) (http://www.cdn.ca).

### DNA extraction and genotypes

Genomic DNA of the whole blood was extracted using the TIANamp Blood Genomic DNA Purification kit (Tiangen inc. Beijing, China). The criteria of DNA quality control were DNA concentration should be larger than 50 ng/μL, the ratio of OD260/OD280 in the range of 1.7–1.9 and the ratio of OD260/OD230 in the range of 1.5–2.1.

The cows were genotyped using Illumina Bovine SNP50 BeadChip [25]. The genotypes were edited according to the criteria: (1) call rate > = 90 %; (2) SNPs did not deviated extremely from Hardy-Weinberg equilibrium (P >10^−6^); (3) minor allele frequency > = 3 %). After quality control, a total of 43,885 SNPs were available for MMRA. Distribution of SNPs on each chromosome after quality control and the average distances between adjacent SNPs are shown in Additional file 1: Table S1.

### Association analysis

Mixed model based single locus regression analysis (MMRA) applied to perform GWAS in our studies is as follows:

MMRA:

$$ \mathbf{y}=\upmu + b\mathrm{x} + \mathrm{Z}\mathrm{a} + \mathrm{e} $$

Where *y* is the vector of phenotypes (SCS EBVs), *μ* is the overall mean, *b* is the vector of coefficients of the regression on SNP genotypes, **x** is the vector of SNP genotypes, *a* ~ (0, Aσ_a_^2^) and *e* ~ (0, Wσ_e_^2^) are the vectors of the polygenic effects and residuals, where A is the additive genetic relationship matrix and W is a diagonal matrix with diagonal elements of 1/REL_i_ to weight residuals variance for heterogeneity [26]. REL_i_ is the reliability of EBV for the i^th^ individual. σ_a_^2^ and σ_e_^2^ is the additive variance and residual error variance respectively. For each SNP, the estimated *b* and $Var\left(\widehat{b}\right)$ are obtained via mixed model equations (MME). In addition, an approximate Wald chi-squared statistic ${\widehat{b}}^2/Var\left(\widehat{b}\right)$ with *df =1* is estimated for the SNPs significantly associated with phenotypes. This association analysis was conducted using a program written in FORTRAN language by our group [26].

### Statistical inference

To decrease the false positive rate of multiple tests and screen more available SNPs as well as find more functional related genes, Bonferroni multiple testing (*P* < 0.05) was adopted to adjust for number of SNPs on genome and chromosome level. The results of Bonferroni threshold for genome and each chromosome divided by 0.05 were listed in Additional file 2: Table S2.

Linkage disequilibrium analysis for the significant SNPs on BTA 14 was performed using Haploview software (version 4.2) [27].

Student *t*-tests were conducted to compare the difference of cows SCS EBVs with different genotypes in each candidate gene.

## Additional files

Additional file 1: Table S1. Distribution of SNPs on each chromosome after quality control and the average distances between adjacent SNPs. These data were derived from Bos_taurus_UMD_3.1 assembly (http://www.ncbi.nlm.nih.gov/assembly/GCF_000003055.4/). SNPs which are not assigned to any chromosomes are noted as “0”. (DOCX 25.5 kb) Additional file 2: Table S2. Results of Bonferroni thresholds at genome-wide level and at chromosome-wide level for each chromosome. SNPs which are not assigned to any chromosomes are noted as “0”. (DOCX 24.8 kb)

## Abbreviations

GWAS: Genome-wide association study
SNP: Single nucleotide polymorphism
SCC: Somatic cell count
SCSs: Somatic cell scores
EBVs: Estimated breeding values
BTA: *Bos taurus* autosome
MMRA: Mixed model based single locus regression analysis
*LY6*: Lymphocyte-antigen-6 complex
*TLR4*: Toll-like receptor 4
*BRCA1*: Breast cancer 1
*TRAPPC*9: Trafficking protein particle complex 9
*ARHGAP39*: Rho GTPase activating protein 39
LD: Linkage disequilibrium
DIM: Days in milk
GO: Gene Ontology
NIBP: NIK and IKKβ-binding protein
NIK: NF-κB-inducing kinase
IKKβ: IκB kinase-β
ROADTRIPs: Robust Association-Detection Test for Related Individuals with Population Substructure
IACUC: Institutional Animal Care and Use Committee
CDN: Canadian Dairy Network
MME: Mixed model equations
