# Supplementary material for: Genome-wide association study in Chinese Holstein cows reveal two candidate genes for somatic cell score as an indicator for mastitis susceptibility
Source: BMC Genet. 2015 Sep 15;16:111. doi: 10.1186/s12863-015-0263-3 (PMC4570044; doi:10.1186/s12863-015-0263-3)
Supplement: Additional file 1: Table S1. — Distribution of SNPs on each chromosome after quality control and the average distances between adjacent SNPs. These data were derived from Bos_taurus_UMD_3.1 assembly (http://www.ncbi.nlm.nih.gov/assembly/GCF_000003055.4/). SNPs which are not assigned to any chromosomes are noted as “0”. (DOCX 25.5 kb) [file 12863_2015_263_MOESM1_ESM.docx]

**Additional file: Table S1.** Distribution of SNPs on each chromosome after quality control and the average distances between adjacent SNPs^1^

| Chr. | No. SNPs | Average distance (kb) | Chr. | No. SNPs | Average distance (kb) |
| --- | --- | --- | --- | --- | --- |
| 1 | 2743 | 57.72 | 17 | 1307 | 57.50 |
| 2 | 2207 | 62.10 | 18 | 1113 | 59.30 |
| 3 | 2106 | 57.66 | 19 | 1147 | 55.85 |
| 4 | 2050 | 58.94 | 20 | 1325 | 54.37 |
| 5 | 1751 | 69.21 | 21 | 1126 | 63.59 |
| 6 | 2082 | 57.38 | 22 | 1039 | 59.13 |
| 7 | 1825 | 61.72 | 23 | 906 | 57.98 |
| 8 | 1945 | 58.30 | 24 | 1047 | 59.90 |
| 9 | 1646 | 64.22 | 25 | 816 | 52.58 |
| 10 | 1762 | 59.20 | 26 | 883 | 58.53 |
| 11 | 1862 | 57.63 | 27 | 816 | 55.65 |
| 12 | 1356 | 67.23 | 28 | 782 | 59.22 |
| 13 | 1443 | 58.38 | 29 | 862 | 59.75 |
| 14 | 1398 | 60.55 | X | 556 | 267.67 |
| 15 | 1382 | 61.72 | 0 | 1310^2^ |  |
| 16 | 1292 | 63.25 | Total | 43885 |  |

^1^Derived from Bos_taurus_UMD_3.1 assembly (http://www.ncbi.nlm.nih.gov/assembly/GCF_000003055.4/);

^2^These SNPs are not assigned to any chromosomes.
