# Supplementary material for: Genome-wide association study in Chinese Holstein cows reveal two candidate genes for somatic cell score as an indicator for mastitis susceptibility
Source: BMC Genet. 2015 Sep 15;16:111. doi: 10.1186/s12863-015-0263-3 (PMC4570044; doi:10.1186/s12863-015-0263-3)
Supplement: Additional file 2: Table S2. — Results of Bonferroni thresholds at genome-wide level and at chromosome-wide level for each chromosome. SNPs which are not assigned to any chromosomes are noted as “0”. (DOCX 24.8 kb) [file 12863_2015_263_MOESM2_ESM.docx]

**Additional file: Table S2.** Results of Bonferroni thresholds at genome-wide level and at chromosome-wide level for each chromosome.

| Chr. | Significant *P*-values | Chr. | Significant *P*-values |
| --- | --- | --- | --- |
| 1 | 1.82E-05 | 17 | 3.83E-05 |
| 2 | 2.27E-05 | 18 | 4.49E-05 |
| 3 | 2.37E-05 | 19 | 4.36E-05 |
| 4 | 2.44E-05 | 20 | 3.77E-05 |
| 5 | 2.86E-05 | 21 | 4.44E-05 |
| 6 | 2.40E-05 | 22 | 4.81E-05 |
| 7 | 2.74E-05 | 23 | 5.52E-05 |
| 8 | 2.57E-05 | 24 | 4.78E-05 |
| 9 | 3.04E-05 | 25 | 6.13E-05 |
| 10 | 2.84E-05 | 26 | 5.66E-05 |
| 11 | 2.69E-05 | 27 | 6.13E-05 |
| 12 | 3.69E-05 | 28 | 6.39E-05 |
| 13 | 3.47E-05 | 29 | 5.80E-05 |
| 14 | 3.58E-05 | X | 8.99E-05 |
| 15 | 3.62E-05 | 0 ^1^ | 3.82E-05 |
| 16 | 3.87E-05 | genome-wide | 1.14E-06 |

^1^SNPs which are not assigned to any chromosomes are noted as “0”.
